# Development of a prediction model of conversion to Alzheimer’s disease in people with mild cognitive impairment: the statistical analysis plan of the INTERCEPTOR project

**DOI:** 10.1186/s41512-024-00172-6

**Published:** 2024-07-25

**Authors:** Flavia L. Lombardo, Patrizia Lorenzini, Flavia Mayer, Marco Massari, Paola Piscopo, Ilaria Bacigalupo, Antonio Ancidoni, Francesco Sciancalepore, Nicoletta Locuratolo, Giulia Remoli, Simone Salemme, Stefano Cappa, Daniela Perani, Patrizia Spadin, Fabrizio Tagliavini, Alberto Redolfi, Maria Cotelli, Camillo Marra, Naike Caraglia, Fabrizio Vecchio, Francesca Miraglia, Paolo Maria Rossini, Nicola Vanacore, the INTERCEPTOR Network, Maurizio Belfiglio, Cristina Muscio, Davide Quaranta, Emanuele Cassetta, Mario Barbagallo, Carlo Gabelli, Simona Luzzi, Fulvio Lauretani, Innocenzo Rainero, Carlo Ferrarese, Orazio Zanetti, Michela Marcon, Flavio Mariano Nobili, Giuseppe Pelliccioni, Sabina Capellari, Elena Sinforiani, Gioacchino Tedeschi, Carmen Gerace, Laura Bonanni, Sandro Sorbi, Lucilla Parnetti

**Affiliations:** 1 National Centre for Disease Prevention and Health Promotion, Italian National Institute of Health, Rome, Italy; 2 National Center for Drug Research and Evaluation, Italian National Institute of Health, Rome, Italy; 3 Department of Neuroscience, Italian National Institute of Health, Rome, Italy; 4 Neurology Department and Brain Health Service, Fondazione IRCCS San Gerardo Dei Tintori, Monza, Italy; 5 Department of Biomedical, Metabolic, and Neural Sciences, University of Modena and Reggio Emilia, Modena, Italy; 6 University Institute of Advanced Studies IUSS Pavia, Pavia, Italy; 7 IRCCS Mondino Foundation, Pavia, Italy; 8 Vita-Salute San Raffaele University, Nuclear Medicine Unit, IRCCS San Raffaele Scientific Institute, Milan, Italy; 9 “Associazione Italiana Malattia di Alzheimer” – AIMA, Milan, Italy; 10 Fondazione IRCCS Istituto Neurologico “Carlo Besta”, Milan, Italy; 11 Laboratory of Neuroinformatics, IRCCS Istituto Centro San Giovanni Di Dio Fatebenefratelli, Brescia, Italy; 12 Neuropsychology Unit, IRCCS Istituto Centro San Giovanni Di Dio Fatebenefratelli, Brescia, Italy; 13 Department of Neuroscience, Catholic University of Sacred Heart, Rome, Italy; 14 Memory Clinic, Foundation Policlinico Agostino Gemelli IRCCS, Rome, Italy; 15 Brain Connectivity Laboratory, Department of Neuroscience and Neurorehabilitation, IRCCS San Raffaele, Rome, Italy; 16 Department of Theoretical and Applied Sciences, eCampus University, Novedrate, Como, Italy

**Keywords:** Statistical analysis plan, Longitudinal study, Mild cognitive impairment, Dementia, Alzheimer’s disease, Biomarker, Prediction model

## Abstract

**Background:**

In recent years, significant efforts have been directed towards the research and development of disease-modifying therapies for dementia. These drugs focus on prodromal (mild cognitive impairment, MCI) and/or early stages of Alzheimer’s disease (AD). Literature evidence indicates that a considerable proportion of individuals with MCI do not progress to dementia. Identifying individuals at higher risk of developing dementia is essential for appropriate management, including the prescription of new disease-modifying therapies expected to become available in clinical practice in the near future.

**Methods:**

The ongoing INTERCEPTOR study is a multicenter, longitudinal, interventional, non-therapeutic cohort study designed to enroll 500 individuals with MCI aged 50–85 years. The primary aim is to identify a biomarker or a set of biomarkers able to accurately predict the conversion from MCI to AD dementia within 3 years of follow-up. The biomarkers investigated in this study are neuropsychological tests (mini-mental state examination (MMSE) and delayed free recall), brain glucose metabolism ([^18^F]FDG-PET), MRI volumetry of the hippocampus, EEG brain connectivity, cerebrospinal fluid (CSF) markers (p-tau, t-tau, Aβ1-42, Aβ1-42/1–40 ratio, Aβ1-42/p-Tau ratio) and APOE genotype. The baseline visit includes a full cognitive and neuropsychological evaluation, as well as the collection of clinical and socio-demographic information. Prognostic models will be developed using Cox regression, incorporating individual characteristics and biomarkers through stepwise selection. Model performance will be evaluated in terms of discrimination and calibration and subjected to internal validation using the bootstrapping procedure. The final model will be visually represented as a nomogram.

**Discussion:**

This paper contains a detailed description of the statistical analysis plan to ensure the reproducibility and transparency of the analysis. The prognostic model developed in this study aims to identify the population with MCI at higher risk of developing AD dementia, potentially eligible for drug prescriptions. The nomogram could provide a valuable tool for clinicians for risk stratification and early treatment decisions.

**Trial registration:**

ClinicalTrials.gov NCT03834402. Registered on February 8, 2019

**Supplementary Information:**

The online version contains supplementary material available at 10.1186/s41512-024-00172-6.

## Introduction

### Background and rationale

Over the last two decades, significant efforts have been dedicated to the development of potentially disease-modifying treatments for Alzheimer’s disease (AD) [1, 2]. In recent years, phase 3 clinical trials have been completed on monoclonal antibody therapies proposed to slow down the conversion of AD, and requests for marketing authorisation have been submitted to regulatory authorities. Lecanemab was approved by the Food and Drugs Administration (FDA) in July 2023 and is currently under assessment by the European Medicines Agency (EMA). Aducanumab has received accelerated approval from the FDA for the treatment of the early stage of AD, but it has not been approved in Europe. Donanemab is headed for approval in the USA [3]. These monoclonal antibodies target amyloid plaques in the brain and are intended for those with MCI and mild AD. The MCI population has an estimated 15% incidence of dementia within 2 years, making them at higher risk for developing dementia [4]. However, this population also includes individuals who will never convert to AD, ranging from 14 to 38% depending on the study setting [4, 5]. As monoclonal antibody therapies are expensive and have non-negligible adverse effects [6], selectively targeting individuals with MCI who are at a higher risk of converting to AD dementia is needed. In this context, the Italian Ministry of Health and the Italian Medicines Agency (AIFA) initiated the INTERCEPTOR project in 2018 (https://www.interceptorproject.com) with the primary aim of evaluating the most reliable biomarker or set of biomarkers for predicting the conversion from MCI to AD dementia within a 3-year follow-up period. The study is designed as a longitudinal cohort study, where the baseline clinical and biomarker characteristics of the individuals diagnosed with MCI will be analysed in relation to their risk of conversion to AD dementia. The findings of this study will be used to identify individuals eligible for potential disease-modifying treatment in clinical practice. The secondary aim of the project is to define an optimal organisational model that can be readily implemented in clinical practice aligning with the primary goal and being economically sustainable. However, this aim is not part of the present statistical analysis plan.

### Study objectives

The primary aim of the study is to develop and internally validate a multivariable prediction model capable of identifying a biomarker or a set of biomarkers to predict the conversion from MCI to Alzheimer’s disease after 3 years of follow-up.

## Methods

The analyses outlined in this document are in full compliance with the TRIPOD statement for Transparent Reporting of the development of a multivariable prediction model for Individual Prognosis Or Diagnosis [7] (see Additional file 1). The statistical analysis plan has been finalised prior to the completion of the data collection. The study was registered at www.clinicaltrials.gov (NCT03834402) in January 2019. Details on the study protocol have been published elsewhere [8]. The analyses will be carried out using Stata v17.0 (StataCorp, College Station, Texas, USA) and R software v4.3.0 (R Foundation for Statistical Computing, Vienna, Austria).

### Study design

This is a multicentre, interventional, non-therapeutic cohort study. A sample of 500 individuals consecutively diagnosed with MCI at the Center for Cognitive Disorders and Dementia (Centri per i Disturbi Cognitivi e Demenza, CDCD) was planned to be enrolled. The follow-up process, consisting of neuropsychological and clinical assessments, is scheduled every 6 months over a 36-month observation period. Recruitment started on December 21, 2018, and the follow-up of the last recruited person will end on December 22, 2023.

### Study population

The study includes individuals of any gender, aged 50 to 85 years, with a formal diagnosis of MCI according to the National Institute on Aging-Alzheimer’s Association (NIA-AA) criteria [9]. The exclusion and inclusion criteria are provided in Table 1. Participants newly diagnosed with MCI were consecutively recruited from 19 Italian memory clinics (CDCD), which are centres with documented expertise in the diagnosis and treatment of AD and MCI, distributed across the national territory in each country’s macro-areas (Northwest, North East, Central, South, Islands) to reflect a representative reproduction of the Italian Health System organisation in this field. Each recruiting centre performed a comprehensive neuropsychological evaluation, collected blood samples for genetic testing, performed lumbar puncture for cerebrospinal fluid (CSF) analysis and acquired an electroencephalogram (EEG), FDG-PET scan and brain magnetic resonance imaging (MRI) either on site or at facilities associated with the recruiting centres. The evaluation of each biomarker and the risk group classification were carried out by four expert centres of reference selected for their expertise. Additional details regarding the organisational architecture of the project, protocol implementation and exam acquisition are reported elsewhere [8].

**Table 1:** Inclusion and exclusion criteria

**Inclusion criteria:**
a) Age between 50 and 85 years
b) Age and education corrected mini-mental state examination score equal or superior to 24/30
c) Clinical Dementia Rating (CDR) global score of 0.5
d) Concerns about cognitive modifications, expressed as subjective complaints by the subject, or by impression by a close acquaintance or an expert clinician
e) Defective performance with reference to age and education matched controls in one cognitive domain (memory, executive function, attention, language, visuospatial function): if repeated assessments are available, evidence of performance decline
f) Preserved functional autonomy: the subject remains fully independent, even if specific performances may be slower, less efficient than usual level, with occasional errors
g) No dementia: the cognitive modifications do not significantly hamper social function or work activities
**Exclusion criteria:**
a) History of cerebrovascular disease (i.e. stroke episodes), alcohol abuse, severe medical disorders associated with cognitive impairment (organ failures, endocrine disorders, in particular thyroid disease and B12/folates deficiency); neuroimaging evidence of other potential causes of cognitive decline (e.g. subdural haematoma, malignancy); chronic treatment with psychotropic drugs; women in reproductive age
b) History of malignancy < 5 years
c) Contraindications for magnetic resonance imaging (MRI): pacemaker; spinal stimulators; defibrillator; any other condition incompatible with MRI acquisition
d) Presence of spinal malformations or any other contraindications to lumbar puncture, according to the investigator’s judgement
e) HIV infection
f) Use of drugs potentially affecting cognitive function, according to the investigator’s judgement
g) Individuals are not allowed to participate in any trial with experimental drug
**Exclusion criteria specific to lumbar puncture:**
Patients who refuse to or cannot temporarily interrupt antiplatelet or anticoagulant therapy 14 days prior to sampling visit

#### Baseline characteristics

Participants will be characterised based on all variables collected at baseline, separately for those who will convert to dementia and those who will not. These variables include socio-demographic information, medical history, physical examination, risk factors, comorbidities and concomitant medications (Additional file 2). Categorical data will be summarised by absolute frequencies and percentages. Continuous data will be summarised using mean and standard deviation for normally distributed variables and median with interquartile range for skewed distributions. Statistical comparisons will be performed using *t*-tests, Wilcoxon rank-sum tests and chi-square tests as appropriate. Statistical significance will be set at the 5% level.

### Outcomes

#### Primary outcome

The primary outcome is the conversion to AD dementia within 3 years of follow-up after the diagnosis of MCI. Visits to assess the study outcome are planned every 6 months. Follow-up time starts at the date of neuropsychological evaluation at baseline and ends at (i) date of conversion, (ii) end of follow-up, (iii) death and (iv) loss to follow-up, whichever occurs first. The conversion to dementia will be diagnosed by specialists according to the American Psychiatric Association’s Diagnostic and Statistical Manual of Mental Disorders, Fifth Edition (DSM-5) on the basis of clinical, cognitive and functional examination, including the CDR. The diagnosis will not be performed based on any of the biomarkers under study as clinicians are blinded with respect to the biomarkers evaluation which is a necessary condition to correctly evaluate the predictive performance of biomarkers and avoid biased estimation of the association between predictors and outcome. Conversion to Alzheimer’s disease is confirmed when an individual receives two consecutive diagnoses of the same type. The date of the first diagnosis will be considered as the date of conversion.

#### Secondary outcome

The secondary outcomes are the conversion to Alzheimer’s disease within 3 years of follow-up in amnesic patients and the conversion to other forms of dementia.

### Predictors

Biomarkers were selected for their accuracy in predicting the conversion from MCI to AD dementia based on the best evidence available in the literature [8]. The biomarkers under investigation include the Mini-Mental State Examination (MMSE), Delayed Free Recall (DFR) as a subscore of the Free and Cued Selective Reminding test (FCSRT), cerebrospinal fluid (CSF) parameters, (^18^F)FDG-PET, volumetric MRI, EEG for brain connectivity and APOE genotype. Details on the standardisation procedure for acquiring each biomarker are provided in Rossini et al. [8].

For CSF biomarkers, the following fluid levels are considered: threonine-181 (p-tau), total-tau (t-tau), amyloid-beta Aβ1-42, Aβ_1-42/1–40_ ratio, Aβ_1-42_/p-tau ratio. ^18^Fluorodeoxyglucose positron emission tomography (^18^F)FDG-PET is analysed with voxel-based single subject using the optimised procedure based on statistical parametrical mapping (SPM), involving a large database of normal controls for comparison and the new FDG atlas for normalisation [10, 11]. Two techniques are used to compute the right and left hippocampal volumes, normalised to the intracranial volume in volumetric MRI. The first technique is ACM-Adaboost, which is a machine learning algorithm used to classify the voxels belonging to the hippocampal region. The second technique is Freesurfer v6.0, which is a surface-based approach [12]. Seven parameters of Small World (SW) are computed in delta, theta, alpha1, alpha2, beta1, beta2 and gamma frequency bands to evaluate the brain connectivity via graph theory in EEG [13]. As part of the study, each participant is required to undergo all necessary tests for biomarker acquisition. These tests are required to be completed within 60 days from the neuropsychological battery assessment and the acquisition of informed consent at the beginning of the study. Biomarker testing is conducted in a blinded manner, meaning that the data collected at baseline, including predictor variables such as biomarkers and any information related to medical history or clinical examination, are not taken into account during biomarker testing. Moreover, to improve the predictive accuracy of biomarkers, clinical and demographic characteristics will be also considered. A critical review of the relevant literature was conducted to identify factors associated with the development of dementia [14, 15]. The following predictors, selected according to the clinical relevance among all the demographic and clinical variables collected, will be used in the development and validation of the multivariable prediction model: sex, age, level of education (measured in years of education), type of MCI (amnesic, non-amnesic) according to single or multiple domains, Amsterdam IADL (short version) [16], Cumulative Illness Rating Scale (CIRS) including severity and comorbidity index [17], hypertension, family history of dementia, smoking status, status of cohabitation as a proxy of social isolation, psychiatric disease (past and present) and cardiovascular disease. All these data, collected during the clinical assessment visit, could significantly improve the accuracy of the prediction model. All predictors are collected at baseline.

### Sample size

To determine the sample size, the methodological work of Bujang and Adnan [18] was followed. This work provides guidance for estimating the minimum sample size required in a screening or diagnostic study to achieve the desired sensitivity and specificity with adequate power and a controlled level of type I error. Based on the literature review, the level of sensitivity for the biomarkers under study ranges between 52 and 81%, while the specificity ranges between 52 and 86%. After a follow-up of about 2.5 years, the incidence of AD in a cohort of individuals with MCI was observed to range from 29 to 46% [19, 20]. Based on this data and utilising the tables provided by Bujang and Adnan, a minimum sample size of 388 individuals with MCI is needed assuming an expected incidence of AD of 40% after a 3-year follow-up period, a sensitivity value of 80% for an individual biomarker (working hypothesis), a power ≥ 80% and a *p*-value lower than 5%. Additionally, applying the formula described by Buderer [21] which incorporates the event frequency into the sample size calculation, it was verified that 400 evaluated individuals would ensure an acceptable level of precision (the half-width of the 95% CI) between 4 and 7.5% for both sensitivity and specificity (Table 2). Assuming a dropout rate of 20%, a total sample of 500 individuals with MCI has been determined.

**Table 2:** Level of precision^a^ on the estimates of sensitivity and specificity (for a range of values) corresponding to a sample size of 400, according to an incidence of AD ranging from 27 to 40%

Incidence	Sensitivity			Specificity		
	**80%**	**85%**	**90%**	**80%**	**85%**	**90%**
***N* = 400**						
**27%**	7.5%	6.7%	5.7%	4.6%	4.1%	3.4%
**35%**	6.6%	5.9%	5.0%	4.9%	4.3%	3.6%
**40%**	6.2%	5.5%	4.6%	5.1%	4.5%	3.8%

^a^Formula proposed by Buderer was applied

The primary objective of this study is to develop a prediction model including biomarkers in addition to clinical variables. Following the practical guidance provided by Riley et al. [22] to calculate the number of events needed for developing a multivariable prediction model, and assuming an expected C-index of 0.8 [23] which corresponds to an apparent Cox-Snell *R*^2^ of 0.31, and considering an overall incidence rate of 16% (based on an average follow-up of 2.5 years), we have determined that the number of events required per predictor parameter is 9.5. Consequently, the planned sample is deemed sufficient to include up to 17 candidate predictors.

### Statistical analysis method

All the analysis will be conducted on patients with the baseline assessment of biomarkers available; consequently, patients without biomarker assessment will be excluded from the primary analysis.

To exclude selection bias, a comparison of baseline characteristics of individuals not included in the analysis due to incomplete biomarkers assessment and those included in the analysis will be performed.

A multivariable predictive model of conversion to AD will be developed including biomarkers, socio-demographic and clinical predictors. The evaluation of biomarkers for MMSE, DFR, CSF parameters and MRI measures will be conducted considering the continuous form and the categorical form applying validated cut-offs.

For the MMSE [24], a cut-off of 24 (< 24) for the age- and sex-corrected score will be used, as indicated in the literature [25]. For the correction of the crude score, Measso et al. [26] will be used for individuals aged ≤ 64, and Magni et al. [25] will be used for those aged > 64. Regarding the DFR, a test result will be classified as abnormal if the age-adjusted score is ≤ 6.31 [27].

For MRI, measurements of the right and left hippocampus are considered for each technique, ACM-Adaboost and Freesurfer, and atrophy is defined below the 5th percentile according to the age-specific thresholds defined for individuals aged 56–90 years, as published [12]. For individuals aged 50–55 years, the cut-offs are extrapolated from the 5th percentile function (see Additional file 2). Right and left hippocampal atrophy measured with the two techniques will be analysed separately.

For CSF biomarkers, p-tau, t-tau, Aβ1-42, Aβ_1-42/1–40_ ratio and Aβ_1-42_ /p-tau ratio will be evaluated separately. The cut-offs used for each biomarker are reported in Table 3 along with the literature references [28–31]. For the EEG, the classification of results into normal or abnormal based on the SW values will be performed by the expert centre using a machine learning (ML) method based on fine Gaussian support vector machine. This ML classification model has been trained on external data including both healthy individuals and individuals with AD [32] and will be applied to the study cohort. The FDG-PET result will be categorised as either positive or negative by the evaluators at the expert centre based on the methodology previously described [10, 11]. In the case of FDG-PET positivity, it will also be specified whether it is indicative of an AD-like form or other forms of dementia. The APOE-e4 genotype will be classified as none, one (heterozygosis), or both alleles (homozygosis) (see Table 3 and Additional file 3 for details of the cut-offs and literature references).

**Table 3:** Reference value for identifying abnormal values by biomarkers

Biomarker	Reference value for categorisation	Literature and notes
**MMSE**	Age- and sex-adjusted score < 24	Applied correction factors from Measso et al. [26] for age ≤ 64 and Magni et al. [25] for age > 64
**DFR**	Age-adjusted score ≤ 6.31	Frasson et al. 2011 [27]
**Hippocampal volume**	< 5° percentile of the normative population	De Francesco et al. 2021 [12]; see Additional file 3
**CSF biomarkers**		
Aβ (1–42)	≤ 640 pg/ml	Bellomo et al. 2021 [28]
Total-tau	≥ 404 pg/ml	Bellomo et al. 2020 [29]
Phosphorylated Tau 181 (p-Tau)	≥ 56 pg/ml	Bellomo et al. 2020 [29]
Ratio Aβ_1-42/1–40_	≤ 0.068	Doecke et al. 2018 [30], Leitão et al. 2019 [31]
**Ratio** Aβ_1-42 /p-Tau_	≤ 11.8	Leitão et al. 2019 [31]
**EEG**	Classified in physiological vs pathological based on Small World for the seven EEG frequency bands using machine learning technique	Vecchio et al. 2023 [32]
**FDG-PET**	Classified in negative and positive (AD-like, other forms) using the optimised SPM procedure	Della Rosa et al. 2014 [10], Perani et al. 2014 [11]
**APOE- e4**	Classified in none, one allele, both the alleles	

However, these cut-offs were determined to discriminate between normal cognition and AD, but the population under study is at higher risk of developing dementia compared to the normal population. Therefore, for continuous biomarkers the receiver operating characteristic (ROC) curves will be used to identify new optimal cut-offs for the specific population under study. For biomarkers known to be influenced by sex and/or age, the corrected values will be used. In this context, sensitivity and specificity are both considered targets for determining the optimal cut-off; therefore, among all the commonly used methods [33], the distance to the left-upper corner was chosen. This method maximises both sensitivity and specificity and guarantees a better balance between the two measures [34]. Warnings are raised about its use as not always the method leads to an optimal cut-off in the intention of maximising overall correct classification rates compared to the commonly used Youden’s method [35]; therefore, Youden’s method will be also provided for comparison.

#### Statistical model and variable selection

Initially, an analysis of socio-demographic and clinical variables will be conducted using the Cox model to identify a core of variables significantly associated with the outcome that will be included in the development of the prognostic multivariable model. A check of multicollinearity between predictors will be assessed, then a stepwise selection starting from the full model will be applied using a threshold of 0.10 for removal and 0.05 for re-admission of variables. A final check of variables not selected by stepwise will be conducted using the likelihood ratio test [36, 37]. Age and sex will be forced in the procedure selection and retained in the final model. Subsequently, these selected variables will be included in multivariable models with the addition of individual biomarkers one at a time. The additional predictive value of each biomarker beyond clinical and socio-demographic variables will be calculated. The performance results will be used to determine for each biomarker which form (continuous or categorical) will be chosen for the development of the multivariable prediction model. Categorisation is typically preferred for the application in clinical practice, but as any categorisation implies a loss of information, we will select the biomarker in a categorical form if the loss in performance could be considered negligible with respect to the continuous form. Categorisation will also be chosen in cases where non-linearity is detected for the continuous form. The categorisation according to the threshold provided by the experts (Table 3) will be preferred in case a small loss of performance is observed compared to the continuous form.

The final predictive model will be identified through a multivariable model considering as eligible predictors all the biomarkers and all selected predictors among the clinical and socio-demographic variables using the stepwise selection procedure as described above. Age and sex will be forced into the model selection process. A final check of the result from the automatic selection procedure will be conducted to ensure that no significant biomarkers were excluded and, vice versa, that no negligible biomarkers were included.

#### Checking assumptions and variable handling

The relationship between study outcome and each continuous predictor will be explored. Linearity will be tested. Transformation or categorisation of variables will be considered for dealing with non-linearity. Collinearity between predictors will be measured with the variance inflation factors (VIF) [38, 39]. Values equal to 1 indicate that variables are not correlated, VIF values greater than 5 are generally considered a cause of concern, and values exceeding 10 are indicative of problematic multicollinearity that needs to be corrected. The proportional hazards assumption will be tested using Schoenfeld residuals and checked by visual inspection of the log–log plot. If the assumption is not satisfied, the variable by time interaction will be included in the model.

#### Predictive performance

For each biomarker in the categorical form, the accuracy measures (sensitivity, specificity, positive predictive value, negative predictive value, overall accuracy, positive and negative likelihood ratios) will be calculated for comparison with the literature. For continuous biomarkers, the area under the receiver operating characteristic curve (AUC) will be also reported. Moreover, a univariate analysis will be conducted using the proportional hazard Cox model.

To evaluate the performance of the prediction models, several measures will be considered. The concordance c-index with its 95% CI will be provided as a discrimination measure and the extensions of binary AUC to survival analysis proposed by Harrell will be used [40, 41]. The Akaike information criterion will be provided as a measure of the goodness of fit of the model.

The added value on performance of each biomarker to the multivariable model with demographical and clinical variables will be assessed by the category-free Net Reclassification Improvement (NRI), which is the net proportion of events reclassified correctly plus the net proportion of non-events reclassified correctly. The delta AUC (difference in c-statistics) will be also provided [41], and the likelihood ratio test will be used to assess the statistical significance of the incremental contribution of each biomarker [36, 37].

In addition, to assess the predictive performance of the final multivariable model, calibration measures will also be reported [38, 42]. A calibration plot will be done by plotting the observed outcome proportion (on the *y*-axis) versus the ordered predicted outcome probabilities (on the *y*-axis) by quantile of the predicted risk. Subsequently, the calibration intercept and slope and their 95% CI will be estimated. There is a perfect calibration when the intercept is zero and the slope is one. Moreover, overall measures of performance, the Nagelkerke’s *R*^2^ and the Brier score, will be provided. The Brier score is a composite measure of discrimination and calibration with lower scores indicating improved model accuracy, and it will be calculated at a fixed time point (3 years).

### Missing data

To contain the number of missing data and patient dropout, telephone contact with individuals who missed scheduled visits, or their caregivers, was planned offering the option of a remote visit if attendance was not feasible. No missing data will be accepted in the evaluation of biomarkers; therefore, it is required that all participants have a full evaluation of the biomarkers under study. A high level of completeness is expected for the main baseline characteristics, especially for age, sex and level of education. At least 80% of completeness will be required to include predictive factors in the analysis and the multiple imputation techniques by chained equations will be applied for dealing with missing data. Missing values for continuous normally distributed data will be imputed using linear regression, while the predictive mean matching method will be applied for not normally distributed data. Logistic regression will be used for binary data and ordinal logistic for ordered categorical data [43]. All predictors, including socio-demographic and clinical variables and biomarkers, will be considered potential variables for the imputation model. The outcome variable will be included in the imputation model adding the censoring indicator and the cumulative baseline hazard function estimated with the Nelson–Aalen method. For each variable with missing data to be imputed, candidate variables for inclusion in the imputation model will be selected based on their association with the missingness indicator. Associations will be assessed using *t*-test for continuous variables and chi-square test for categorical variables, with a *p*-value threshold of 0.05. If the frequency of missing data is less than 5% for each selected predictive variable, the main analysis will be conducted using only one imputed value generated according to the approach described above using the predictive mean imputation with 5 nearest neighbours to preserve variance and avoid the inflation of associations among the variable and predictors. A complete case analysis will be performed on this imputed dataset.

#### Internal validation

The final predictive model will be internally validated using the bootstrap technique with 500 bootstrap samples [38]. The stepwise selection will be repeated in the validation process (i.e. stepwise procedure will be repeated within each bootstrap sample). Consequently, Harrel’s c-index of the predictive model will be adjusted to take into account the estimated potential overfitting and a corrected c-index will be reported. Apparent and corrected calibration measures will be reported [44]. The correction will be performed using the shrinkage factor calculated with bootstrapping. The shrinkage factor may take values between 0 and 1, and a value above 0.90 indicates a small overfitting. As the shrinkage of regression coefficients is an important way to reduce overfitting, the shrinkage factor will be also applied to regression coefficients and intercept of the final model [38] to improve the prediction ability of the regression model when applied to external data. Both the original and shrunken regression coefficients will be reported.

#### Sensitivity analysis

As sensitivity, the robustness of the stepwise procedure for the selection of variables in the final model will be performed by repeating the stepwise selection considering different levels of p for removal and for re-admission and using the Akaike information criterion (*p* ≤ 0.157). Additionally, the least absolute shrinkage and selection operator (LASSO) method will be used, and the tuning parameter lambda will be chosen by tenfold cross-validation. The predictive performance of the models will be compared.

A complete case analysis will be performed for the final model if the number of missing data will be contained.

Additionally, the competing risk analysis will be performed taking into account death as a competing event using Fine and Gray’s semiparametric proportional sub-distribution hazards model.

### Model presentation and risk groups

For each predictive model, the hazard ratios with 95%CI and *p*-value will be reported. For the final predictive model, a nomogram will be generated. A nomogram is a graphical representation of the statistical predictive model. It is a visual tool that translates model numeric results into a visual format, making it easier to estimate an outcome based on multiple variables. Each variable occurrence is assigned a specific score based on its contribution to the overall prediction. The total score from all variables is then used to estimate the probability of the predicted event (in this case, the conversion to AD at 3 years) conditioning to the individual characteristics [45]. For this purpose, the covariates in the Cox model will be centred. Sensitivity and specificity corresponding to different thresholds of the prediction risk score will also be provided for the final model. Finally, three or more risk categories will be proposed defining low-, medium- and high-risk groups based on the predictive risk score considering the highest sensitivity corresponding to an adequate level of specificity. This category definition will help the clinician with the choice to treat individuals with MCI based on the likelihood of converting to dementia in the following 3 years. Because avoiding the clinical consequence of treating a false positive is a target as important as treating a true positive, a rate of false positives less than 20% (i.e. specificity of at least 80%) is considered acceptable for a medium–high-risk group. We will start by inspecting deciles of the predictive score and grouping them into homogeneous classes based on levels of sensitivity and specificity. Kaplan–Meier curves will be presented for each risk group.

#### Additional analysis

The COVID-19 pandemic occurred while the recruitment was still ongoing. A growing literature has shown the impact of restrictive measures adopted during the pandemic such as isolation and COVID-19 infection on cognitive decline [46–48]. Data regarding COVID-19 infections, severity of COVID-19 leading to hospitalisation and requirement of intensive care unit and vaccination status will be reported. Characteristics of individuals enrolled before and during the pandemic period will be compared to verify whether the pandemic had an impact on the recruitment of participants. Furthermore, the association of COVID-19 infection will be evaluated by univariate and multivariable Cox model including the status variable as time-dependent.

#### Analysis of secondary clinical endpoints

For secondary clinical endpoints, conversion to AD in amnesic patients and conversion to other forms of dementia, univariate analysis will be repeated. The final multivariable predictive model identified in the primary analysis will be applied. The model performance will be assessed by Harrel’s C-index, as well as the other performance measures specified in the primary analysis.

## Discussion

To our knowledge, the INTERCEPTOR study represents the first publicly funded study to evaluate, through a harmonisation process among specialised centres distributed throughout the Italian territory, a wide set of biomarkers aimed at predicting the conversion from MCI to AD based over an adequate period of follow-up. The strength of this study lies in the inclusion of a broad range of biomarkers and the standardised and homogeneous collection of these biomarkers. Substantial efforts have been made to standardise biomarker acquisition and analysis, with specialised centres providing expertise in risk diagnosis classification [8]. The study has encountered challenges posed by the COVID-19 pandemic, primarily evident in the recruitment slowdown. The COVID-19 pandemic occurred when the study was in the recruitment phase. For patient recruitment, participants needed to undergo multiple visits for clinical assessment and biomarker acquisition, including at clinical centres distinct from the recruitment centre. The pandemic limited access to certain services necessary for clinical visits and biomarker acquisition. The timeline for scheduled visits has been impacted, leading to deviations from the protocol, which will be documented and appropriately addressed. Despite implementing mitigation actions aimed at ensuring the completion of the INTERCEPTOR study, such as extending the recruitment period and introducing remote visits, we were not able to reach the targeted sample size. Although the initial goal of enrolling 500 individuals with MCI was not met, the final sample size is anticipated to provide estimates of accuracy measures with sufficient precision and to enable the development of a predictive model incorporating demographical and clinical information alongside biomarkers. The model will undergo internal validation, enhancing its reliability. Nevertheless, one major limitation of this study is the lack of external validation, which is crucial to assess the generalizability and robustness of the model. However, once the model is defined, a comparison will be made with existing prognostic models for conversion from MCI to AD available in the literature, and the feasibility of conducting an external validation using data provided by other studies will be evaluated. Furthermore, it is important to note that certain methodological decisions, such as handling non-linearity and categorisation of biomarkers, were not strictly guided by the best evidence but were made to prioritise the clinical interpretability and applicability of the model. This highlights a trade-off between model performance and practical utility in clinical settings. The decision to represent the identified predictive model in the form of a nomogram aims to facilitate its interpretability and applicability with the final goal of assisting clinicians in assessing eligibility for prescribing upcoming new treatments proposed for dementia.

## Conclusion

This paper presents the details of the statistical analysis to be conducted in accordance with the relevant guidelines. The results of the pre-specified analyses will be subsequently made available to minimise the outcome reporting bias.

### Supplementary Information

Additional file 1. TRIPOD checklist.

Additional file 2. Variables collected at baseline.

Additional file 3. Age-specific volume thresholds of the 5th percentile for hippocampal volume computed with FreeSurfer and ACM-Adaboost.

## Data Availability

Not applicable.

## Abbreviations

AD: Alzheimer’s disease
AIFA: Italian Medicine Agency
AUC: Area under the receiver operating characteristic curve
APOE: Apolipoprotein E
CDCD: Center for Cognitive Disorders and Dementia
CDR: Clinical Dementia Rating
CI: Confidence interval
CSF: Cerebrospinal fluid
DFR: Delayed free recall
DSM-5: Diagnostic and Statistical Manual of Mental Disorders, Fifth Edition
EEG: Electroencephalogram
EMA: European Medicines Agency
FCRST: Free and Cued Selective Reminding Test
FDA: Food and Drug Administration
FDG-PET: Fluorodeoxyglucose-positron emission tomography
MCI: Mild cognitive impairment
MMSE: Mini-mental state examination
MRI: Magnetic resonance imaging
ML: Machine learning
NIA-AA: National Institute on Aging-Alzheimer’s Association
NRI: Net Reclassification Improvement
ROC: Receiver operating characteristic
SPM: Statistical Parametrical Mapping
SW: Small World
TRIPOD: Transparent Reporting of a multivariable prediction model for Individual Prognosis Or Diagnosis
VIF: Variance inflation factor
