# Supplementary material for: Development of a prediction model of conversion to Alzheimer’s disease in people with mild cognitive impairment: the statistical analysis plan of the INTERCEPTOR project
Source: Diagn Progn Res. 2024 Jul 25;8:11. doi: 10.1186/s41512-024-00172-6 (PMC11271065; doi:10.1186/s41512-024-00172-6)
Supplement: Supplementary file 2 — Additional file 2. Variables collected at baseline. [file 41512_2024_172_MOESM2_ESM.docx]

**Variables collected at baseline**

| **Variables at baseline** | **Description** |
| --- | --- |
| **Socio-demographic characteristics** | |
| Age | Years |
| Gender | M, F |
| Education | Years |
| Level of education | None, primary school,  middle school, high school,  degree, postgraduate |
| Marital status | Single, married  widower, separated/divorced |
| Co-habitation | Nobody, partner, children  relative (sister, brother, mother, father…), other |
| Smoking status | Non-smoker, ex-smoker, currently smoker |
| **Clinical parameters** | |
| BMI | kg/m^2^ |
| Blood pressure | mmHg |
| Total cholesterol | mg/dL |
| Hypertension | No, yes |
| Cardiovascular disease | No, yes |
| Psychiatric disease | No, yes |
| **Medical history** | |
| Family history for dementia | None, mother, father, other relatives |
| Family history for psychiatric disorder | No, yes |
| Previous psychiatric disease | No, yes |
| Previous episodes of delirium | No, yes |
| **MCI diagnosis** | |
| Type of MCI | Amnesic, non-amnesic |
| Domain | Single, multiple |
| Time from symptoms onset | Years |
| **Concomitant medication** | |
| Any current medication:  cardiological  nervous systems  other | No, yes  No, yes  No, yes |
| Type of medication | ATC categories, second level |
| **Clinical and neuropsychological assessment** | **Short description and range** |
| Cumulative Illness Rating Scale (CIRS)^1^  Severity index  Comorbidity index | 13 items corresponding to organ system and assessing illness severity and comorbidity  Severity index: average of all items (range 1-5)  Comorbidity index: count of organ systems with moderate or greater impairment (range 0-13) |
| Sleep disorders (PEQOL)^2^ | 5 items questionnaire investigating sleep disorders (range 0-25) |
| Neuropsychiatric Inventory (NPI)^3^ | 10 items corresponding to behavioural domains, (range 0-120) |
| Clinical Dementia Rating Scale (CDR)^4^ | 6 categories used for rating dementia severity  (range 0-3) |
| Amsterdam IADL- Short version^5^ | 30 items investigating differences in instrumental activities of daily living (range 0-120) |
| Free and Cued Selective Reminding Test (FCSRT)^6^  Immediate Free Recall (IFR)  Immediate Total Recall (ITR)  Delayed Free Recall (DFR)  Delayed Total Recall (DTR)  Index of sensitivity of Cueing (ISC) | Memory test controlling attention and acquisition, divided into the following subscore:  IFR (range 0-36)  ITR (range 0-36)  DFR (range 0-12)  DTR (range 0-12)  ISC (range 0-1) |
| Mini Mental State Examination (MMSE)^7^ | 11 items assessing cognitive function (range 0-30) |
| Rey’s Auditory Verbal Learning Test (RAVLT)^8^  Recall  Delayed recall  Recognition | 30 items divided in two lists assessing verbal recall and recognition. Recall is repeated over 5 trials, Delayed recall and recognition over 1 trial each (range: 0–15 for each trial) |
| Raven’s coloured progressive matrices  (CPM47)^9^ | 36 items test assessing visuoperceptual, language, visual and verbal memory (range 0-36) |
| Trail Making Test (TMT)^10^  A  B | TMT A and TMT B assessing visual attention and processing speed.  Time in seconds required for completion |
| Rey-Osterrieth complex figure (ROCF)^11^  Copy  Delayed recall | Test assessing visuospatial abilities, memory, attention, planning, and working memory consisting of two different trials: Copy and Delayed recall (range 0-36 for each trial) |
| Verbal fluency test^12^  Phonemic  Semantic | Varying number of items assessing spontaneous verbal production based on letter fluency (Phonemic) or category fluency (Semantic) |

ATC=Anatomical Therapeutic Chemical

^1^ Parmelee PA, Thuras PD, Katz IR and Lawton MP. Validation of the Cumulative Illness Rating Scale in a Geriatric Residential Population. Journal of the American Geriatrics Society. 1995; 43: 130-137. https://doi.org/10.1111/j.1532-5415.1995.tb06377.x

^2^ De Leo D, Frisoni GB, Rozzini R, Bernardini M, Dello Buono M and Trabucchi M. (1991) The Profile of Elderly Quality of Life (PEQOL): A quick package to assess general health conditions in old age. 5th Congress of the International Psychogeriatric Association, Rome.

^3^ Cummings JL, Mega M, Gray K, Rosenberg-Thompson S, Carusi DA, Gornbein [The Neuropsychiatric Inventory: comprehensive assessment of psychopathology in dementia.](https://pubmed.ncbi.nlm.nih.gov/7991117/) J.Neurology. 1994 Dec;44(12):2308-14. doi: 10.1212/wnl.44.12.2308.PMID: 7991117

^4^ Morris JC. [The Clinical Dementia Rating (CDR): current version and scoring rules.](https://pubmed.ncbi.nlm.nih.gov/8232972/) Neurology. 1993 Nov;43(11):2412-4. doi: 10.1212/wnl.43.11.2412-a.

^5^ Jutten RJ, Peeters CF, Leijdesdorff SM, Visser PJ, Maier AB, Terwee CB, et al. Detecting functional decline from normal aging to dementia: Development and validation of a short version of the Amsterdam IADL Questionnaire. Alzheimers Dement (Amst). 2017 Mar 31;8:26-35. doi: 10.1016/j.dadm.2017.03.002. eCollection 2017.PMID: 28462387

^6^ Frasson P, Ghiretti R, Catricalà E, Pomati S, Marcone A, Parisi L, Rossini PM, Cappa SF, Mariani C, Vanacore N, Clerici F. Free and cued selective reminding test: an Italian normative study. Neurol Sci. 2011 Dec;32(6):1057-62. doi: 10.1007/s10072-011-0607-3. Epub 2011 May 19. PMID: 21594655

^7^ Folstein MF, Folstein SE, McHugh PR. “Mini Mental State”. A practical method for grading the cognitive state of patients for the clinician. J Psychiatr Res. 1975 Nov;12(3):189-98. doi: 10.1016/0022-3956(75)90026-6.

^8^ Caltagirone C, Gainotti G, Carlesimo CA, Parnetti L, & il Gruppo per la standardizzazione della Batteria per il Deterioramento Mentale (1995). Batteria per la valutazione del deterioramento mentale (parte I): descrizione di uno strumento di diagnosi neuropsicologica. Archivio di psicologia, Neurologia e Psichiatria, 4, 461-470.

^9^ Basso A, Capitani E, Laiacona M. Raven’s Coloured Progressive Matrices: normative values on 305 adult normal controls. Funct Neurol. 1987 Apr-Jun;2(2):189-94.PMID: 3666548.

^10^ Giovagnoli AR, Del Pesce M, Mascheroni S, Simoncelli M, Laiacona M, Capitani E. Trail Making Test: normative values from 287 normal adult controls. Ital J Neurol Sci. 1996 Aug;17(4):305-9. doi: 10.1007/BF01997792.

^11^ Cafarra P, Vezzadini G, Dieci F, Zonato F, Venneri A. Rey-Osterrieth Complex Figure: Normative values in an Italian population sample. Neurol Sci. 2002 Mar;22(6):443-7. doi: 10.1007/s100720200003.PMID: 11976975.

^12^ Novelli G, Papagno C, Capitani E, Laiacona M, Vallar G, Cappa SF. (1986). Tre test clinici di ricerca e produzione lessicale. Taratura su soggetti normali. Archivio di Psicologia Neurologia e Psichiatria, 4(47):477-506
