# Supplementary material for: Development of a prediction model of conversion to Alzheimer’s disease in people with mild cognitive impairment: the statistical analysis plan of the INTERCEPTOR project
Source: Diagn Progn Res. 2024 Jul 25;8:11. doi: 10.1186/s41512-024-00172-6 (PMC11271065; doi:10.1186/s41512-024-00172-6)
Supplement: Supplementary file 3 — Additional file 3. Age-specific volume thresholds of the 5th percentile for hippocampal volume computed with FreeSurfer and ACM-Adaboost. [file 41512_2024_172_MOESM3_ESM.docx]

**Age-specific volume thresholds of the 5^th^ percentile for hippocampal volume computed with FreeSurfer and ACM-Adaboost**

|  | Adaboost | | FreeSurfer | |
| --- | --- | --- | --- | --- |
|  | LEFT | RIGHT | LEFT | RIGHT |
| Age |  |  |  |  |
| *50* | 4713.7 | 5049.2 | 3311.1 | 3644.2 |
| *51* | 4692.0 | 5020.0 | 3296.2 | 3612.2 |
| *52* | 4670.3 | 4990.7 | 3281.4 | 3580.4 |
| *53* | 4648.5 | 4961.4 | 3266.7 | 3548.8 |
| *54* | 4626.7 | 4932.1 | 3252.0 | 3517.5 |
| *55* | 4605.0 | 4902.6 | 3237.4 | 3486.4 |
| 56 | 4583.2 | 4873.2 | 3222.8 | 3455.6 |
| 57 | 4561.3 | 4843.6 | 3208.2 | 3424.9 |
| 58 | 4539.5 | 4814.0 | 3193.2 | 3394.3 |
| 59 | 4517.6 | 4784.4 | 3177.5 | 3363.6 |
| 60 | 4495.8 | 4754.7 | 3160.8 | 3332.8 |
| 61 | 4473.9 | 4725.0 | 3142.7 | 3301.6 |
| 62 | 4452.0 | 4695.3 | 3123.0 | 3269.8 |
| 63 | 4430.0 | 4665.4 | 3101.8 | 3237.3 |
| 64 | 4408.1 | 4635.5 | 3079.1 | 3204.2 |
| 65 | 4386.1 | 4605.5 | 3055.0 | 3170.4 |
| 66 | 4364.2 | 4575.4 | 3029.4 | 3135.6 |
| 67 | 4342.2 | 4545.3 | 3002.2 | 3099.9 |
| 68 | 4320.1 | 4515.1 | 2973.3 | 3063.2 |
| 69 | 4298.1 | 4484.9 | 2942.5 | 3025.7 |
| 70 | 4276.0 | 4454.7 | 2909.3 | 2987.3 |
| 71 | 4254.0 | 4424.7 | 2873.8 | 2948.3 |
| 72 | 4231.9 | 4394.7 | 2837.2 | 2909.3 |
| 73 | 4209.8 | 4364.9 | 2800.3 | 2870.8 |
| 74 | 4187.6 | 4335.2 | 2764.0 | 2833.3 |
| 75 | 4165.5 | 4305.7 | 2728.7 | 2796.8 |
| 76 | 4143.3 | 4276.2 | 2694.7 | 2761.5 |
| 77 | 4121.1 | 4246.9 | 2662.8 | 2727.7 |
| 78 | 4098.9 | 4217.6 | 2633.6 | 2695.4 |
| 79 | 4076.7 | 4188.4 | 2607.7 | 2664.9 |
| 80 | 4054.5 | 4159.3 | 2585.8 | 2636.2 |
| 81 | 4032.2 | 4130.2 | 2568.2 | 2609.5 |
| 82 | 4009.9 | 4101.2 | 2554.6 | 2584.5 |
| 83 | 3987.6 | 4072.3 | 2544.2 | 2560.9 |
| 84 | 3965.3 | 4043.4 | 2536.1 | 2538.4 |
| 85 | 3942.9 | 4014.6 | 2529.4 | 2516.8 |
| 86 | 3920.6 | 3985.8 | 2523.6 | 2495.7 |
| 87 | 3898.2 | 3956.9 | 2518.4 | 2475.1 |
| 88 | 3875.8 | 3928.1 | 2513.8 | 2454.9 |
| 89 | 3853.4 | 3899.2 | 2509.6 | 2435.0 |
| 90 | 3830.9 | 3870.2 | 2505.7 | 2415.3 |

Note. For ages 50-54 years, the cut-offs are extrapolated from the 5^th^ percentile function
